# The effect of escitalopram in treating mild to moderate depressive disorder and improving the quality of life in patients undergoing coronary artery bypass grafting – a double-blind randomized clinical trial

**DOI:** 10.3389/fpsyt.2024.1342754

**Published:** 2024-06-28

**Authors:** Abdolvahab Baradaran, Mohammad Reza Khodaie Ardakani, Fatemeh Sadat Bateni, Fatemeh Asadian-Koohestani, Mohsen Vahedi, Afsaneh Aein, Nazila Shahmansouri, Gita Sadighi

**Affiliations:** ^1^ Cardiovascular Department of Firouzabadi Hospital, Iran University of Medical Sciences, Tehran, Iran; ^2^ Psychosis Research Center, University of Social Welfare and Rehabilitation Sciences, Tehran, Iran; ^3^ Substance Abuse and Dependence Research Center, University of Social Welfare and Rehabilitation Sciences, Tehran, Iran; ^4^ Department of Health Promotion and Education, School of Public Health, Tehran University of Medical Sciences, Tehran, Iran; ^5^ Psychosomatic research center, Tehran University of Medical Sciences, Tehran, Iran

**Keywords:** escitalopram, depression, quality of life, coronary artery surgery, coronary artery disease

## Abstract

**Introduction:**

Chronic depression and anxiety can be a risk factor for coronary aArtery bypass grafting (CABG) and is an emerging factor after coronary artery disease when the patient is admitted to the hospital and after surgery. We aimed to assess the effect of Escitalopram in treating mild to moderate depressive disorder and improving the quality of life in patients undergoing CABG.

**Methods:**

In this randomized clinical trial, 50 patients undergoing CABG referred to Tehran Heart Hospital from January 2021 to May 2021 and were suffering from mild to moderate depression were randomly assigned to one of the two groups of Escitalopram or placebo. The level of depression was assessed based on Beck’s depression inventory and the quality-of-life status and its domains were assessed based on the SF-36 questionnaire in 2 groups. Measurements were obtained at baseline and at four and eight weeks after treatment. Chi-square, Fisher’s exact, paired, and Wilcoxon tests or ANOVA were used as appropriate.

**Results:**

There was no significant difference between the level of depression between the two study groups at baseline (P=0.312). There was no significant difference between the quality of life and its domains in the two study groups at baseline (P=0.607). However, the most important effect of Escitalopram was reducing depression scores in the intervention group at weeks 4 and 8 after treatment compared to the placebo group (P<0.001). The quality of life and its domains were significantly higher in the Escitalopram group eight weeks after treatment (P=0.004). The amount of drug side effects at 2 and 4 weeks after treatment had no significant difference between the groups (P>0.05).

**Conclusion:**

Escitalopram was effective in treating mild to moderate depressive disorder and improving quality of life in patients undergoing CABG.

**Clinical trial registration:**

https://irct.behdasht.gov.ir/, identifier IRCT20140126016374N2.

## Introduction

Currently, 35% of all deaths in Iran are caused by cardiovascular diseases (1, 2). Coronary Artery Bypass Graft (CABG) is one of the common treatments for patients with coronary artery stenosis (3). In addition to the fact that this method has an important role in alleviating the pain and suffering of patients and increasing survival (4), it also leads to multi-dimensional improvement that includes the physical and mental status as well as the quality of life of the person (5, 6).

Depression and coronary artery disease (CAD) have a mutual relationship, on the one hand, CAD can cause depression in the patient, and on the other hand, depression is an independent risk factor for CAD (7, 8). Depression and anxiety are risk factors for the development and progression of CAD and can facilitate the incidence of CAD as a risk factor through various mechanisms such as heart rhythm disorders, metabolic disorders, water and electrolyte disorders, increased blood pressure, delayed wound healing, increased risk of infection, and impairing performance and effective adaptation (9, 10).

Also, the rate of symptoms of anxiety and depression is high before and after the operation among these patients (11). Depression is present in 20-25% of patients even one year after CABG and is used as an independent diagnostic factor for predicting morbidity, mortality, and cardiovascular problems after CABG in these patients (12, 13). Studies have reported depression prevalence of 20%, and 28% in these patients 10 days after surgery (14, 15). In a study conducted on 155 patients undergoing CABG, it was shown that patients who were depressed before the operation had higher anxiety and depression after the operation (16). The mortality rate in depressed patients after CABG was two to three times higher compared with the group without postoperative depression (17).

Quality of life is a multidimensional concept that includes physical, psychological, and social conditions. Health-related quality of life is a reflection of the effects of the disease and its treatment according to the patient’s perspective and experiences. Various factors affect the quality of life, among which we can mention physical and mental health. In people with CAD, surgery interferes with the normal course of life (15). In patients undergoing CABG, the change in the quality of life is one of the important consequences of this procedure (18). Researchers believe that only performing CABG does not lead to an improvement in the quality of life of people after the operation, but the control of anxiety and stress before and immediately after the operation plays an important role in improving people’s quality of life in the long term (19, 20).

Concerning the high prevalence of depression following CABG, and the importance of early identification and treatment of depression with therapeutic interventions in early stages (21, 22), a medicine that causes minimal cardiac and hemodynamic complications, and at the same time has better effectiveness and less interference with other drugs is preferable for the treatment of heart patients undergoing CABG (23–26). In this regard, serotonin reuptake inhibitors (including Escitalopram) seem to be effective and with limited side effects (27–29).

A study on 369 patients with major depressive disorder and acute coronary syndromes has shown that sertraline is effective and safe for the treatment of depression in these patients (30).

On the other hand, escitalopram has allosteric properties compared to other SSRIs and is more effective in treating depression compared to sertraline and paroxetine (31).

Since many drugs are used in patients with cardiac complications, and in many cases, there are multiple underlying diseases such as diabetes mellitus and hypertension, in this study, Escitalopram was preferred because of fewer side effects, better tolerance, and less interference.

In most studies investigating the effects of antidepressants on cardiac patients, the patients were already taking antidepressants, commonly SSRIs, before undergoing CABG Surgery (32, 33). In a meta-analysis, it has been reported that the use of SSRIs before surgery was not associated with increased bleeding after CABG but had led to an increased need for blood transfusions in patients (34).

Therefore, we aimed to assess the effect of Escitalopram in treating mild to moderate depressive disorder and improving the quality of life in patients after having a CABG.

## Patients and methods

**Figure 1:**
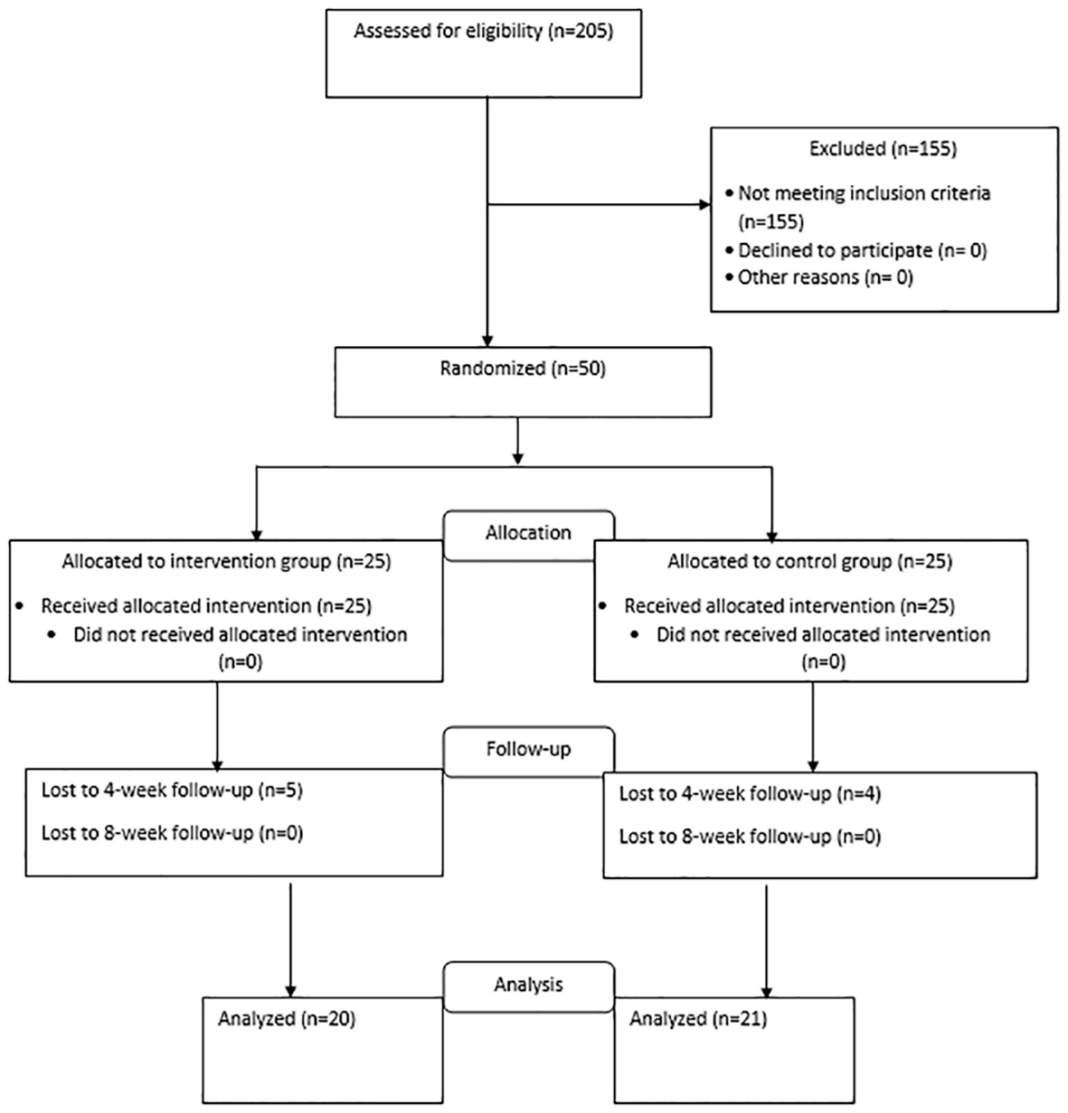
CONSORT flow diagram for participant enrollment.

This study is a double-blind randomized clinical trial. Taking into account the sample size formula based on the study of Bagherian and colleagues (25), 50 patients who are candidates for CABG who visited the Tehran Heart Center Hospital between January 1, 2021, and May 1, 2021, and underwent surgery were enrolled. The samples were randomly assigned to the intervention or control group based on a computer-generated sequence of 25 people in each group. The intervention group is the group that received Escitalopram in addition to the usual cardiovascular treatment, and the control group only received the usual cardiovascular treatment along with a placebo. **Figure 1** shows the flow diagram for patient enrollment and group allocation. Simple randomization based on the computer randomization method was done before conducting the study. Every patient who met the inclusion criteria of the study was allocated randomly to one of the intervention or control groups based on the Excel Rand function.

The inclusion criteria were as follows: CABG, 18-75 years of age, signing the written informed consent form, patients with a Beck Depression Inventory (BDI) score of 10 to 20, and being able to read and write. We excluded participants with a history of SSRI tolerance, those with severe and life-threatening medical conditions that prevented the patient from participating during the intervention (such as emergency CABG, high risk of cardiac complications after surgery such as bleeding and arrhythmia, QT prolongation, delirious state), patients with severe depression and suicidal thoughts who needed any immediate treatment and were referred and treated outside the study, cognitive heart failure diagnosed by a cardiologist, long-term treatment with anticoagulants, severe liver, kidney or thyroid disease, those who participated in other trials, history of bipolar disorder, patients who experience severe complications with Escitalopram (Escitalopram contraindications), patients who have been treated with Escitalopram or antidepressant drugs in the previous month, and recent consumption of alcohol and drugs. This clinical trial study is registered in the Iranian Registry of Clinical Trials (IRCT ID: IRCT20140126016374N2) and the trial protocol can be accessed on the IRCT website.

## Ethical considerations

Written informed consent was obtained from the participants before data collection. They were informed that their information would remain confidential and that they could leave the study any time they wished. No cost was imposed. The protocol of the study was approved by the Ethics Committee of the University of Social Welfare and Rehabilitation Sciences (Code: IR.USWR.REC 1399.159). Moreover, the patient’s health was more important than anything else, so if there was a situation in the study that required the patient’s doctor to know the type of treatment of the patient, this information was provided. In this study, in case of emergency or serious side effects, the main researcher referred the patient to the supervisor of the project and after clinical examinations, it was decided to withdraw him from the study and change the treatment protocol. If during the implementation of the project, due to emergency conditions, there was a need for decoding, this was done only by the supervisor, and the rest of the research team remained blind to the groups.

## Procedure

The initial psychological questionnaires were done when the patients were stabilized in terms of their cardiac status and were ready to answer the questions (five days after surgery and at the time of discharge). A psychiatric interview was also conducted.

Demographic characteristics including age, sex, marital status, place of residence, level of education, history of physical problems, history of psychiatric diseases and depression, and history of drug abuse were asked. Patients were evaluated through a questionnaire, and patients who had a BDI score of 10-20 confirmed by a psychiatric assistant based on a clinical interview and the diagnostic and statistical manual of DSM5 mental disorders were enrolled. Then, the status of their quality of life was also examined through the SF-36 quality of life questionnaire.

In addition to the usual cardiovascular treatment, the participants of the intervention group received Escitalopram 10 mg daily (5 mg for the first week) manufactured by Abidi Company (Iran) and underwent this treatment for 8 weeks. The control group was treated and followed for 8 weeks only with the usual cardiovascular treatment and a placebo, which was completely similar to the original drug manufactured by the same company. The patients were visited at the end of the 2nd and 4th week and were examined in terms of drug side effects, and if any side effect occurred, it was recorded in the drug side effect questionnaire. Also, the BDI was completed for all patients at the beginning of the study, and after four and eight (immediately after the completion of treatment) weeks by a trained person and a psychiatric assistant. Moreover, at the beginning of the study and the end of week eight, the SF-36 questionnaire was completed and according to this questionnaire Depression and quality of life were recorded for patients.

Both the participants and researchers who were involved in the data collection and analysis were blinded to the type of treatment. The type of treatment allocated to the two groups was completely unpredictable. Allocation to the groups was hidden from the researchers and patients by coding the drug and placebo groups and the generation of the random allocation sequence, enrollment of participants, and assignment of participants to interventions was done by a psychologist. The evaluation of the studied subjects was done by an evaluator outside the research team. The main drug and placebo, which were similar (in terms of smell, color, shape, and taste), were placed in similar cans of the same weight by someone outside the research team and were coded into two groups A and B, and numbered based on a random sequence. Each patient has a unique code. Based on the coding of the drug, the researcher provided the drug to the participant. All patients in the control and intervention groups received allocated intervention (**Figure 1**).

## 36-item short form quality of life questionnaire

This questionnaire has 36 questions that evaluate people’s health in eight different areas. In the scoring of this questionnaire, physical function is evaluated by 10 questions, role disorder due to physical health by 4 questions, body pain by 2 questions, general health by 5 questions, fatigue or vitality by 4 questions, social functioning by 2 questions, role disorder due to emotional health by 3 questions and emotional well-being by 5 questions. The reliability and validity of the Persian translation of this questionnaire have been confirmed in Iran, and its internal correlation was 0.87 based on Cronbach’s alpha (35).

## Beck depression inventory

The second edition of this scale is the revised form of the Beck Depression Questionnaire, which was developed to measure the severity of depression and is more consistent with DSM-4 and covers all the domains of depression based on the cognitive theory of depression. This questionnaire consists of 21 groups of questions that ask the respondents to rate the severity of the symptoms from 0 to 3 to show the intensity of the person’s feelings. Therefore, the total score ranges from 0 to 63. This scale has been widely used in Iran, and its psychometric properties have been confirmed. For example, Dobson and Mohammadkhani reported a test-retest reliability of 0.93 and showed its convergent validity in the correlation between the scores of the BDI with the scores of the Beck Hopelessness Scale, suicidal thoughts, and the Hamilton Depression Scale (36). Ghasemzadeh et al. reported Cronbach’s alpha of 0.87 for internal consistency, a correlation coefficient of 0.74 for test-retest reliability, and a correlation coefficient of 0.77 with the negative spontaneous thoughts questionnaire (37). This questionnaire can be used in the population of 13 years and above, and its cut-off points are as follows: no depression (0 to 9), mild depression (10 to 16), moderate depression (20-17), and severe depression (21-30). and very severe (<30) (37).

The collected data were analyzed using SPSS software, version 21. Independent t- or U-Mann-Whitney statistical tests, repeated-measure analysis of variance test, paired t-test or Wilcoxon test, and Chi-square test were used as appropriate. The results for quantitative variables were expressed as mean and standard deviation (mean ± SD) and for categorical qualitative variables as percentages. A P<0.05 was considered statistically significant. All ethical considerations were applied in this study and informed consent was obtained from all patients or their legal guardians to participate in the study.

## Results

**Figure 2:**
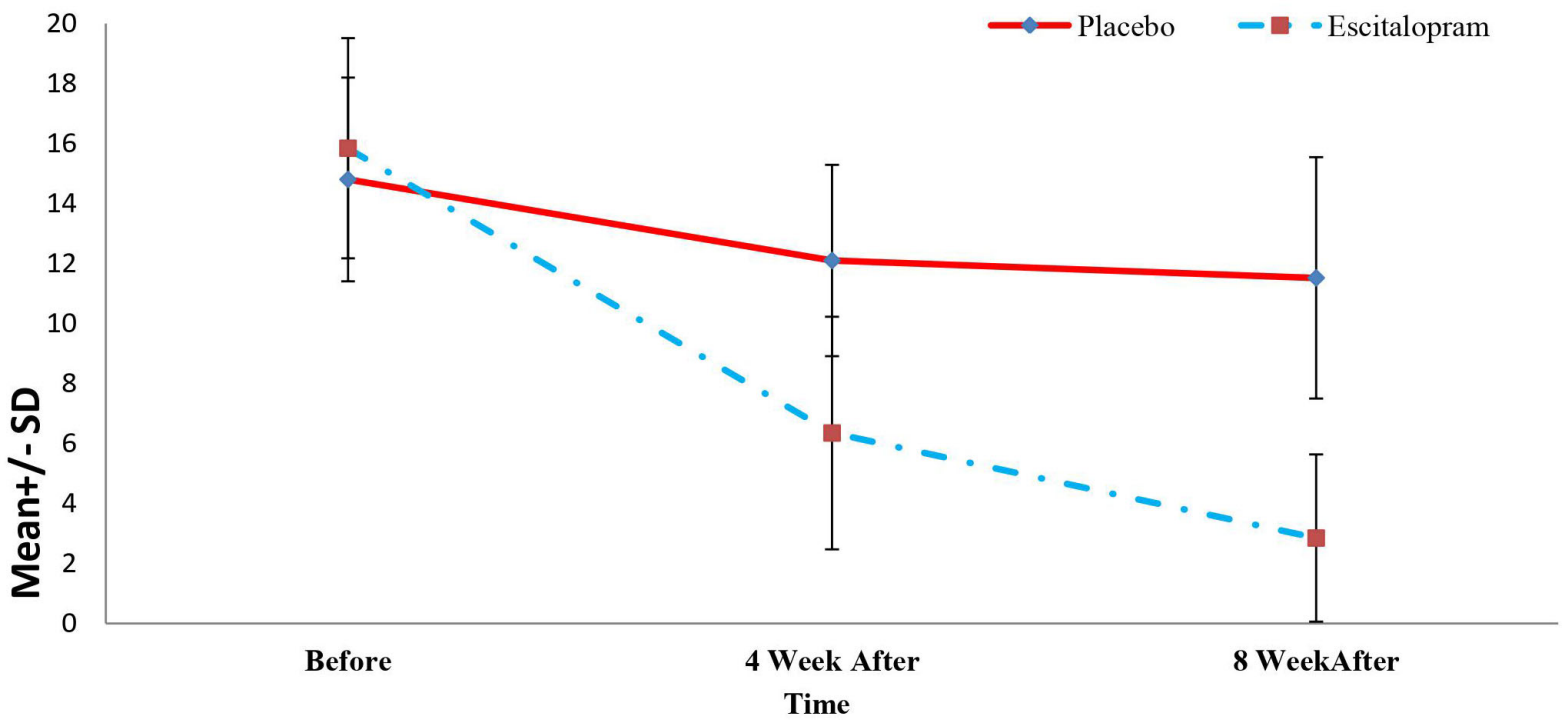
Mean Beck’s depression rating scale scores of patients in two study groups before, 4 and 8 weeks after treatment.

In this study, of the 205 patients who met the conditions to enter the study, 50 patients (24.39%) with a Beck Depression score of 10 to 20 were enrolled in the study. The descriptive characteristics and comparison of demographic characteristics and clinical history of the patients in the two study groups are summarized in **Table 1**. As shown, we found no significant difference between the two groups concerning age, sex, education level, income, employment status, marital status, history of blood pressure, history of hyperlipidemia, history of diabetes mellitus, history of smoking, and psychiatric history of patients (P>0.05, **Table 1**). Mean Beck’s Depression Rating Scale Scores of patients in two study groups before, 4 and 8 weeks after treatment is visible in **Figure 2**.

**Table 1:** Descriptive characteristics and comparison of demographic characteristics and clinical history of patients in the two study groups.

Variable	Group		P value
	Placebo	Escitalopram	
Age (year)			
Standard deviation ± Mean	8.47 ± 57.2	7.95 ± 60.16	0.29
Sex			
Male female	18 (72%) 7 (28%)	18 (72%) 7 (28%)	1
Education			
Highschool Diploma Higher education	15 (60%) 7 (28%) 3 (12%)	14 (58%) 9 (36%) 2 (8%)	0.785
Job-status			
Employed unemployed Retired homemaker	14 (58%) 1 (4%) 3 (12%) 77 (28%)	11 (44%) 1 (4%) 6 (24%) 7 (28%)	0.715
Income level			
Low Middle high	2 (8%) 19 (76%) 4 (16%)	4 (16%) 14 (56%) 7 (28%)	0.326
marital status			
Single Married Divorced widowed	1 (4%) 22 (88%) 1 (4%) 1 (4%)	0 (0%) 21 (84%) 1 (4%) 3 (12%)	0.568
History of Blood pressure			
Yes No	14 (56%) 11 (44%)	12 (48%) 13 (52%)	.778
History of hyperlipidemia			
Yes No	2 (8%) 23 (92%)	3 (12%) 22 (88%)	0.637
History of diabetes			
Yes No	11 (44%) 14 (56%)	17 (68%) 8 (32%)	0.154
Smoking history			
Yes No	9 (36%) 16 (64%)	4 (16%) 21 (84%)	0.196
History of psychiatric disorders and referral to a psychiatrist			
Yes No	2 (8%) 23 (92%)	4 (16%) 21 (84%)	0.667

**Figure 3:**
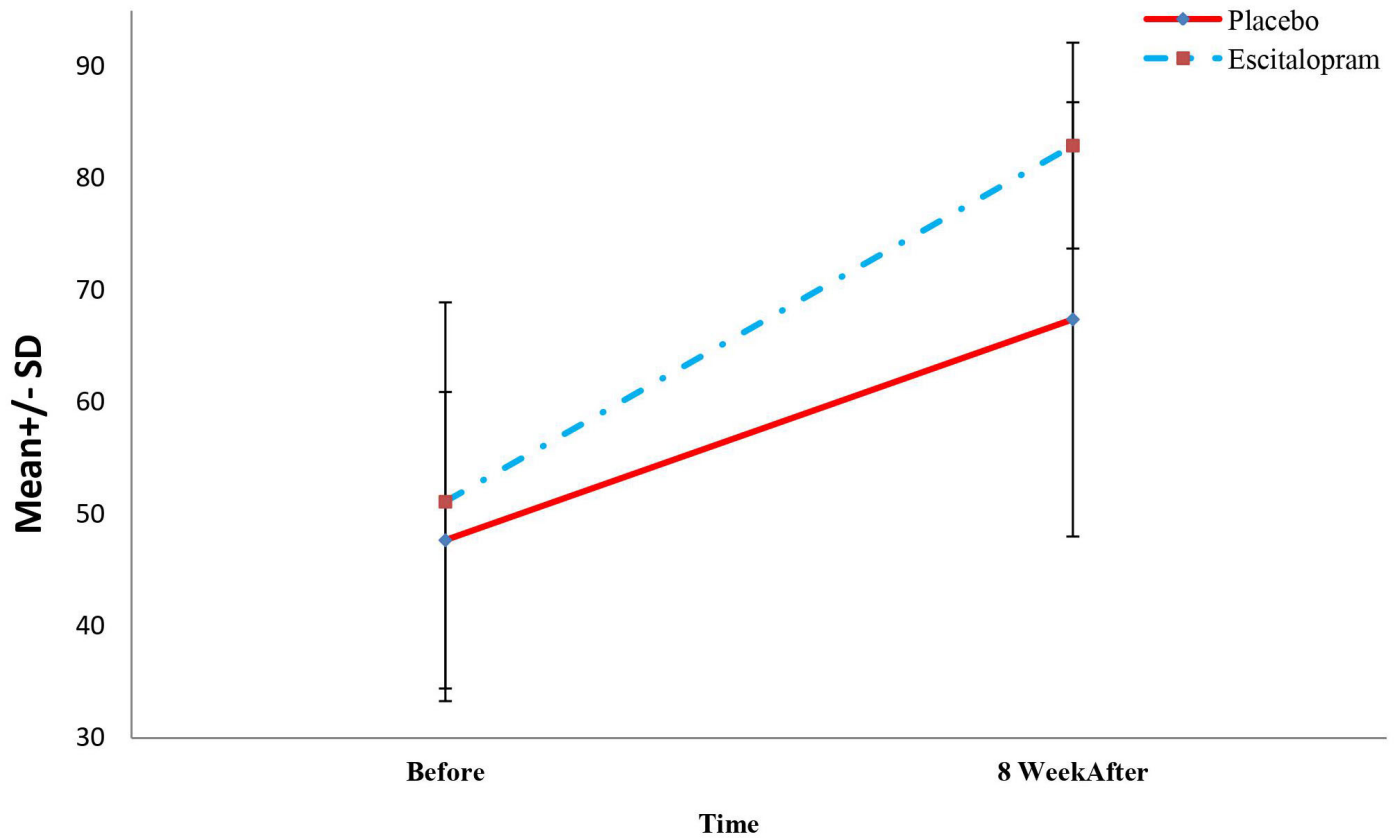
Mean Quality of life questionnaire scores of patients in two study groups before, 4 and 8 weeks after treatment.

The changes in the level of depression of the patients in the study group in the periods before, 4 weeks, and 8 weeks after the treatment were determined and compared with independent t or Mann-Whitney U tests and repeated-measure analysis of variance. The results are summarized in **Table 2**. As shown, inter-group comparisons revealed no significant difference between the level of depression at baseline between the two groups (P=0.312). However, the level of depression 4 and 8 weeks after the treatment was significantly lower in the intervention groups compared with the placebo group (P<0.001). Also, in the intra-group comparisons, it was found that the depression level of the patients in the intervention group decreased significantly during the treatment (P<0.001) and the level of depression of patients in the placebo group did not decrease significantly during the intervention (P=0.08). Mean Quality of Life Questionnaire Scores of patients in two study groups before, 4 and 8 weeks after treatment is visible in **Figure 3**.

**Table 2:** Comparison of the changes in Beck’s depression score of patients in the two study groups before, 4 and 8 weeks after treatment.

Measurement Time	Group		P_1__value
	Placebo Standard ± mean deviation	Escitalopra Standard ± mean deviation	
Before treatment	3.4 ± 14.8	3.67 ± 15.84	0.312
4 weeks after treatment	3.19 ± 12.1	3.88 ± 6.35	<0.001
8 weeks after treatment	4.02 ± 11.52	2.79 ± 2.85	<0.001
**P_2__value**	0.08	<0.001	

To compare the changes in quality of life and its domains between the study groups before treatment and 8 weeks after treatment, independent t or Mann-Whitney U test and paired t or Wilcoxon tests were used. The results are shown in **Table 3**.

**Table 3:** Comparison of the changes in the quality of life and its domains in the patients of the two study groups before and 8 weeks after the treatment.

Domains	Measurement Time	Group		P value
		Placebo Standard ± Mean deviation	Escitalopram Standard ± Mean deviation	
Physical function	Before treatment	32.54 ± 46.4	24.03 ± 51.2	0.591
	8 weeks after treatment	13.25 ± 47.68	17.82 ± 51.12	0.989
	**P_2__value**	<0.001	<0.001	
Physical problems	Before treatment	37.13 ± 24	38.51 ± 24	0.991
	8 weeks after treatment	42.67 ± 57.14	27.47 ± 88.75	0.006
	**P_2__value**	0.017	<0.001	
Physical pain	Before treatment	23.26 ± 47.56	25.08 ± 50.16	0.785
	8 weeks after treatment	20.05 ± 74.19	9.55 ± 83.5	0.108
	**P_2__value**	0.005	<0.001	
General health	Before treatment	19.72 ± 55.44	20.5 ± 59.6	0.468
	8 weeks after treatment	19.57 ± 63.66	13.34 ± 80.1	0.002
	**P_2__value**	0.103	<0.001	
Vitality	Before treatment	15.81 ± 54	18.47 ± 53.2	0.87
	8 weeks after treatment	17.75 ± 56.42	12.61 ± 70.25	0.014
	**P_2__value**	0.475	<0.001	
Social function	Before treatment	22.55 ± 56.24	18.15 ± 63.28	0.135
	8 weeks after treatment	22.43 ± 71.09	15.37 ± 80.95	0.19
	**P_2__value**	0.039	0.001	
Mental problems	Before treatment	47.47 ± 37.36	48.12 ± 53.32	0.192
	8 weeks after treatment	44.09 ± 68.23	26.25 ± 91.65	0.039
	**P_2__value**	0.028	0.022	
Mental health	Before treatment	20.51 ± 60.64	16.72 ± 54.56	0.256
	8 weeks after treatment	19.85 ± 63.8	10.66 ± 80.2	0.007
	**P_2__value**	0.467	<0.001	
Physical health subscale	Before treatment	17.01 ± 45.32	17.21 ± 47.56	0.6
	8 weeks after treatment	17.99 ± 67.23	8.64 ± 82.2	0.006
	**P_2__value**	<0.001	<0.001	
Mental health subscale	Before treatment	14.55 ± 52.8	17.84 ± 56.76	0.394
	8 weeks after treatment	20.99 ± 64.47	12.84 ± 80.65	0.003
	**P_2__value**	0.023	<0.001	
Quality of life	Before treatment	13.25 ± 47.68	17.82 ± 51.12	0.607
	8 weeks after treatment	19.41 ± 67.42	9.2 ± 82.95	0.004
	**P_2__value**	<0.001	<0.001	

Based on the results of **Table 3**, inter-group comparisons between the quality of life and its domains including physical function, physical problems, physical pain, general health, vitality, social function, mental problems, mental health, physical health subscale showed no significant difference in the mental health scale of the patients in the two study groups at baseline (P=0.192). However, 8 weeks after the intervention, the overall level of quality of life was significantly higher in the intervention group (P=0.004). Of course, there was no significant difference between the range of physical function in 8 weeks after the surgery between the two studied groups (P=0.989).

Also, in the intra-group comparisons, we found that the level of quality of life and its domains (including physical function, physical problems, physical pain, general health, vitality, social function, mental problems, mental health, physical health sub-scale, and mental health sub-scale) increased significantly in the intervention group from baseline to 8 weeks after treatment (P<0.001) The level of quality of life and domains such as physical function, physical problems, physical pain, social function, mental problems, mental health, physical health scale, and mental health subscale, also increased significantly in the placebo group from baseline to 8 weeks after surgery (P<0.001). However, the vitality and general health domains of patients differed significantly before and 8 weeks after treatment (P<0.05).

The chi-square test was used to compare the amount of drug side effects in both groups at 2 and 4 weeks after treatment. The most common complications were insomnia (16%) and dry mouth (12%), and other complications reported with less prevalence were itching, nausea, and swallowing disorders (8%). Four weeks after treatment, there was no significant difference between the groups in this regard (P>0.05, **Tables 4**, **5**).

**Table 4:** Comparison of drug Side- effects between two groups after 2 weeks of treatment.

Side effects	Level	Group		P value
		Placebo Frequency	Escitalopram Frequency	
Mouth dryness	No	(84%) 21	22 (88%)	0.43
	Yes	4 (16%)	3 (12%)	
Sweating	No	24 (96%)	25 (100%)	0.312
	Yes	(4%) 1	0 (0%)	
Emotional slowness	No	21 (84%)	25 (100%)	0.11
	Yes	4 (16%)	0 (0%)	
Anxiety and Restlessness	No	23 (92%)	25 (100%)	0.235
	Yes	2 (8%)	0 (0%)	
Drowsiness	No	24 (96%)	24 (96%)	0.368
	Yes	1 (4%)	1 (4%)	
Nausea	No	23 (92%)	23 (92%)	0.513
	Yes	2 (8%)	2 (8%)	
Abdominal pain	No	22 (8%)	24 (96%)	0.13
	Yes	3 (12%)	1 (4%)	
Insomnia	No	19 (76%)	21 (84%)	0.741
	Yes	6 (24%)	4 (16%)	
Headache	No	24 (96%)	25 (100%)	0.312
	Yes	1 (4%)	0 (0%)	
Blurred Vision	No	25 (100%)	24 (96%)	0.312
	Yes	0 (0%)	1 (4%)	
Increased Appetite	No	25 (100%)	24 (96%)	0.312
	Yes	0 (0%)	1 (4%)	
Decreased Appetite	No	22 (88%)	24 (96%)	0.492
	Yes	3 (12%)	1 (4%)	
Sexual Dysfunction	No	24 (96%)	25 (100%)	0.312
	Yes	1 (4%)	0 (0%)	
Palpitation	No	24 (96%)	23 (92%)	0.552
	Yes	1 (4%)	2 (8%)	
Light Headedness	No	24 (96%)	25 (100%)	0.312
	Yes	1 (4%)	0 (0%)	
Skin Rushes or Bruisiness	No	25 (100%)	24 (96%)	0.312
	Yes	0 (0%)	1 (4%)	
Itching	No	25 (100%)	23 (92%)	0.49
	Yes	0 (0%)	2 (8%)	
Dysphagia	No	24 (96%)	24 (96%)	1
	Yes	1 (4%)	1 (4%)	
Flu Like Symptoms	No	22 (88%)	23 (92%)	0.364
	Yes	3 (12%)	2 (8%)	

**Table 5:** Comparison of drug Side- effects between two groups after 4 weeks of treatment.

Side effects	Level	Group		P value
		Placebo Frequency	Escitalopram Frequency	
Mouth dryness	No	24 (96%)	25 (100%)	0.312
	Yes	1 (4%)	0 (0%)	
Emotional slowness	No	22 (88%)	25 (100%)	0.235
	Yes	3 (12%)	0 (0%)	
Anxiety and Restlessness	No	21 (84%)	25 (100%)	0.49
	Yes	4 (16%)	0 (0%)	
Nausea	No	24 (96%)	24 (96%)	0.368
	Yes	1 (4%)	1 (4%)	
Constipation	No	25 (100%)	24 (96%)	0.312
	Yes	0 (0%)	1 (4%)	
Diarrhea	No	25 (100%)	24 (96%)	0.312
	Yes	0 (0%)	1 (4%)	
Abdominal pain	No	23 (92%)	25 (100%)	0.49
	Yes	2 (8%)	0 (0%)	
Indigestion	No	25 (100%)	24 (96%)	0.312
	Yes	0 (0%)	1 (4%)	
Insomnia	No	24 (96%)	25 (100%)	0.312
	Yes	1 (4%)	0 (0%)	
Urinary inconsistence	No	25 (100%)	24 (96%)	0.312
	Yes	0 (0%)	1 (4%)	
Sexual Dysfunction	No	23 (92%)	25 (100%)	0.49
	Yes	2 (8%)	0 (0%)	
Weight gain	No	25 (100%)	24 (96%)	0.312
	Yes	0 (0%)	1 (4%)	
Skin Rushes or Bruisiness	No	25 (100%)	24 (96%)	0.312
	Yes	0 (0%)	1 (4%)	
Itching	No	25 (100%)	23 (92%)	0.312
	Yes	0 (0%)	2 (8%)	
Skin Rushes or Bruisiness	No	25 (100%)	24 (96%)	0.312
	Yes	0 (0%)	1 (4%)	
Itching	No	25 (100%)	23 (92%)	0.49
	Yes	0 (0%)	2 (8%)	
Slow movements	No	24 (96%)	25 (100%)	0.312
	Yes	1 (4%)	0 (0%)	
Dysphagia	No	23 (92%)	23 (92%)	0.513
	Yes	2 (8%)	2 (8%)	
Flu like symptoms	No	25 (100%)	23 (92%)	0.49
	Yes	0 (0%)	2 (8%)	

## Discussion

The findings of this study showed that the level of depression was significantly lower 4 and 8 weeks after treatment in the patients receiving Escitalopram compared with the placebo group. To the best of our knowledge, no study has been conducted to investigate the effect of using Escitalopram for the treatment of mild to moderate depressive disorder and improvement of quality of life in patients undergoing CABG. To the best of our knowledge, few similar studies have been conducted in treating mild to moderate depressive disorder and improving the quality of life in patients after having a coronary artery bypass graft (CABG), and in most trials, patients received antidepressants before the CABG procedure. However, in some previous studies, the effect of using Escitalopram has been investigated for treating depression in patients with cardiovascular complications (28, 38–41).

In most previous studies, after identifying eligible patients to participate in the research, some of them refrained from continuing to participate in the study. However, in our study, no patients withdrew from participating in the study at this stage and before receiving the allocated intervention, which may be due to the hope of patients for symptom improvement of depression after CABG with the use of antidepressant medication (42–45).

Consistent with our findings, Kim and colleagues found that in patients with depression following acute coronary syndrome, 24 weeks of treatment Escitalopram improved depression symptoms (decrease in Beck questionnaire score) as well as lowered the risk of major adverse cardiac events after an average of 8 years (39).

Another study showed that Escitalopram did not have a clear and specific effect in improving depression and reducing mortality and frequent hospitalizations of patients with chronic systolic heart failure and depression. This result indicates the concept of pathophysiology mechanisms for mood disorders in chronic physical diseases that are less responsive to antidepressant drugs (41). Consistently, in another study assessing the effectiveness of citalopram and Escitalopram in people suffering from depressive disorder, the researchers found that during 6 weeks, Escitalopram was better tolerated and more effective in treating people suffering from major depressive disorder (40). Moreover, another study also showed that treatment with citalopram compared to placebo was significantly effective in depression and quality of life of patients with acute coronary syndrome (46).

The level of quality of life and its domains including physical problems, physical pain, general health, vitality, social function, mental problems, mental health, and physical and mental health subscales were significantly higher in the Escitalopram group eight weeks after treatment. The physical function domain showed no significant difference in the two study groups in eight weeks after treatment which can be attributed to complications and pain caused by surgery and its physical limitations. These findings are similar to the findings of Chocron and colleagues in which the quality of life in the domain of physical pain was significantly improved in the Escitalopram group (38). Also, in another study, a reduction in the number and severity of physical complaints and an improvement in the quality of life of 89% of patients were reported (47).

The level of quality of life and its domains including physical functions, physical problems, physical pain, social function, mental problems mental health, and physical and mental health subscales increase ،significantly before and 8 weeks after the treatment. However, there was no significant difference in domains of general health, and vitality of patients in the placebo group before and 8 weeks after the treatment. These findings are in line with other studies that have shown that heart surgery significantly improves people’s quality of life (48). On the contrary, some studies reported unfavorable quality of life in patients after heart surgery. This inconsistency may be due to the difference in the statistical population, measurement tools, follow-up durations, inclusion criteria such as cardiovascular status, and underlying diseases such as diabetes, etc. Also, various factors such as underlying conditions, prescription drugs, economic conditions, family support, energy reduction fatigue caused by the course of the disease and treatment and surgery, and complications caused by cardiac drugs have an impact on the quality of life of patients (49, 50).

It is important to pay attention to safety measures when prescribing SSRIs, including escitalopram, to patients with heart diseases. A study on the interaction between antidepressants and heart medications has shown an increased risk of bleeding with concurrent use of SSRIs with NOACs or warfarin, as well as an increased risk of prolonging QTc interval and potential interactions between escitalopram and metoprolol (51).

Given the high prevalence of depression in patients with cardiovascular diseases, the prescription of antidepressant medication in these patients is inevitable. However, due to the drug interactions and associated risks mentioned, there is a greater need to focus on the type of antidepressant drug chosen and the potential drug interactions to select the safest drug combination.

In this study, patients who had cardiac issues such as arrhythmia, prolonged QT interval, and depressed beat-to-beat variation and bleeding were excluded from the study. Drug- related side effects, including heart palpitation and Skin Rushes or Bruisiness were evaluated in weeks 2 and 4, and no significant differences were found between the two study groups (**Tables 4**, **5**).

Our study had some limitations. Since it was difficult to get access to patients meeting our inclusion criteria, the sample size in the current study was small and the results are not generalizable to other populations. Another Limitation of this study was the short duration of implementation, which did not allow for the assessment of the long-term effects of escitalopram. It is recommended that future studies be conducted with larger sample sizes and longer follow-up periods. Another limitation of our study was not comparing the two groups in terms of biological effect mediators (i.e. CRP, Heart rate variability, etc). It is suggested that future studies also address these points.

## Conclusion

Antidepressant treatment with Escitalopram was effective in improving the quality of life. This finding showed that the treatment of depression affected the patient’s life satisfaction, the feeling of recovery, the reduction of fear and worry, and the increase of the patient’s social and family interactions which ultimately improved the quality of life. As a result, it is important to improve depression and quality of life in patients after heart surgery.

## Data availability statement

The raw data supporting the conclusions of this article will be made available by the authors, without undue reservation.

## Ethics statement

The studies involving humans were approved by the Ethics Committee of University of Social Welfare and Rehabilitation Sciences (Code: IR.USWR.REC 1399.159). The participants provided their written informed consent to participate in this study.

## Author contributions

AB: Conceptualization, Formal analysis, Investigation, Methodology, Resources, Writing – original draft, Writing – review & editing. MA: Conceptualization, Formal analysis, Methodology, Writing – original draft, Writing – review & editing. FB: Data curation, Investigation, Writing – original draft, Writing – review & editing. FA-K: Data curation, Writing – original draft, Writing – review & editing. MV: Data curation, Writing – original draft, Writing – review & editing. AA: Data curation, Investigation, Writing – review & editing. NS: Data curation, Writing – original draft, Writing – review & editing. GS: Conceptualization, Data curation, Formal analysis, Investigation, Methodology, Project administration, Resources, Writing – original draft, Writing – review & editing.
